# Can Gastric Volume be Accurately Estimated by Ultrasound?

**DOI:** 10.5152/TJAR.2022.21341

**Published:** 2022-06-01

**Authors:** Yücel Gültekin, Özgür Kılıç, Zerrin Özçelik, Şükrü Salih Toprak, Recep Bayram, Oğuzhan Arun

**Affiliations:** 1 Department of General Surgery, Uşak University Faculty of Medicine, Uşak, Turkey; 2 Division of Intensive Care Medicine, Department of Internal Medicine, Ondokuz Mayıs University Faculty of Medicine, Samsun, Turkey; 3 Division of Intensive Care Medicine, Department of Anaesthesiology and Reanimation, Sancaktepe Şehit Prof. Dr. İlhan Varank Training and Research Hospital, İstanbul, Turkey; 4 Department of General Surgery, Konya Beyhekim State Hospital, Konya, Turkey; 5 Department of Radiology, Konya Beyhekim State Hospital, Konya, Turkey; 6 Department of Anaesthesiology and Reanimation, Selçuk University Faculty of Medicine, Konya, Turkey

**Keywords:** Cross-sectional area, gastric volume, ultrasonography

## Abstract

**Objective:**

Knowing the degree of gastric fullness is critical in determining the potential risk of pulmonary aspiration prior to urgent or elective intubation. This study aims to investigate the role of ultrasound in predicting the gastric volume accurately.

**Methods:**

176 patients who underwent upper gastric endoscopy after 12-hour fasting were examined by gastric US. The patients were randomly divided into 6 groups according to the volume of ingested semifluid meal: (1) empty stomach (no volume), (2) 50 mL, (3) 100 mL, (4) 200 mL, (5) 300 mL, and (6) 400 mL. Antral cross-sectional area (CSA) was measured by US after each ingestion.

**Results:**

We found a strong linear correlation between antral CSA and gastric volume up to 200 mL. The diagnostic performance of ultrasound was found to be more powerful in the supine position than in the right lateral position. A new mathematical model was established to predict gastric volume. The threshold value for antral cross-sectional area at risk of pulmonary aspiration was determined as 3.1 cm^2^ by sonographic measurement.

**Conclusion:**

Ultrasonography could be preferred to gastric endoscopy or scintigraphy in terms of non-invasiveness and easiness, although it still merits further investigation.

**Main Points**

- Having a full stomach before intubation increases the risk of aspiration. This study aims to investigate the role of gastric ultrasound in predicting the gastric volume.
  A strong linear correlation was established between cross-sectional area (CSA) of stomach and gastric volume ingested up to 200 mL. Gastric CSA of 3.1 cm2 and above was found to be risky for aspiration.
- Gastric ultrasound can be used as an easy, non-invasive and bedside technique to detect the risk of gastric aspiration.

## Introduction

Gastric content is one of the key causes of pulmonary aspiration risk. The critical stomach volume threshold that increases aspiration risk remains controversial. Experimental and clinical research has accepted a gastric content of ≥0.8-1.5 mL kg^−1^ to be significant in terms of increased pulmonary aspiration risk.^1,2^

Aspiration risk varies depending on the duration of the fasting period. Hence, numerous guidelines recommend certain fasting periods to reduce aspiration risk in patients.^3^ Yet, these rules can be ignored in urgent conditions requiring intubation. There is currently no easy and reliable method or a gold standard test for assessing gastric volume. Research with volunteers and certain patient groups suggests that the gastric volume can be evaluated by measuring the gastric antral cross-sectional area (CSA) using ultrasound.^4,5^

After foods are taken into the stomach, they are mixed with the gastric fluid, forming a stomach content called “chyme.” Nearly 100 mL of fluid can be found in the stomach after fasting.^6^ Previous studies investigating gastric volume using ultrasound have either failed to consider the stomach content after fasting or performed “blind” aspiration using a nasogastric tube. Besides, these studies have often evaluated stomach content after intake of liquid or solid contents.^7,8^ Here, however, gastric contents were emptied by endoscopy before ultrasound examination. We performed evaluations on a chyme-like stomach content by intake of solid–liquid mixture foods, consistent with gastric physiology, and investigated the correlation between gastric volume and antral CSA by ultrasound.

## Methods

The current study was conducted in Konya Beyhekim State Hospital in accordance with the ethical standards of Selçuk University Ethic Committee. The patients who underwent upper gastrointestinal endoscopy (UGE) under elective conditions on an outpatient basis were enrolled. Informed consent was obtained from all participants.

Exclusion criteria were being aged below 18 years or above 70 years, having UGE performed at urgent conditions (i.e., acute upper gastrointestinal bleeding), having poor general condition, history of previous abdominal surgery, presence of diabetes mellitus, or pregnancy.

Patients’ demographic characteristics were recorded, including age, sex, height, weight, and body mass index (BMI). Upper gastrointestinal endoscopy procedure was performed under sedation after 12 hours of fasting. During UGE, stomach contents were aspirated, if present. Once the procedure was completed, the patients were included in the study.

A biscuit and clear apple juice was given to each patient to simulate “chyme,” which is a semifluid mixture of digested food and gastric juice. The patients were stratified into 6 groups regarding the amount of ingested volume: (1) no ingestion, (2) 50 mL (12.5 g biscuit and 37.5 mL fruit juice), (3) 100 mL (25 g biscuit and 75 mL fruit juice), (4) 200 mL (50 g biscuit and 150 mL fruit juice), (5) 300 mL (75 g biscuit and 225 mL fruit juice), and (6) 400 mL (100 g biscuit and 300 mL fruit juice).

One minute after taking the biscuit with apple juice, ultrasonographic images of the gastric CSA were obtained in the supine position with the head raised 30° and then in the right lateral decubitus position. An appropriate view of the stomach antrum was received using real-time ultrasound (Aplio 300; Toshiba).

**Figure 1.**
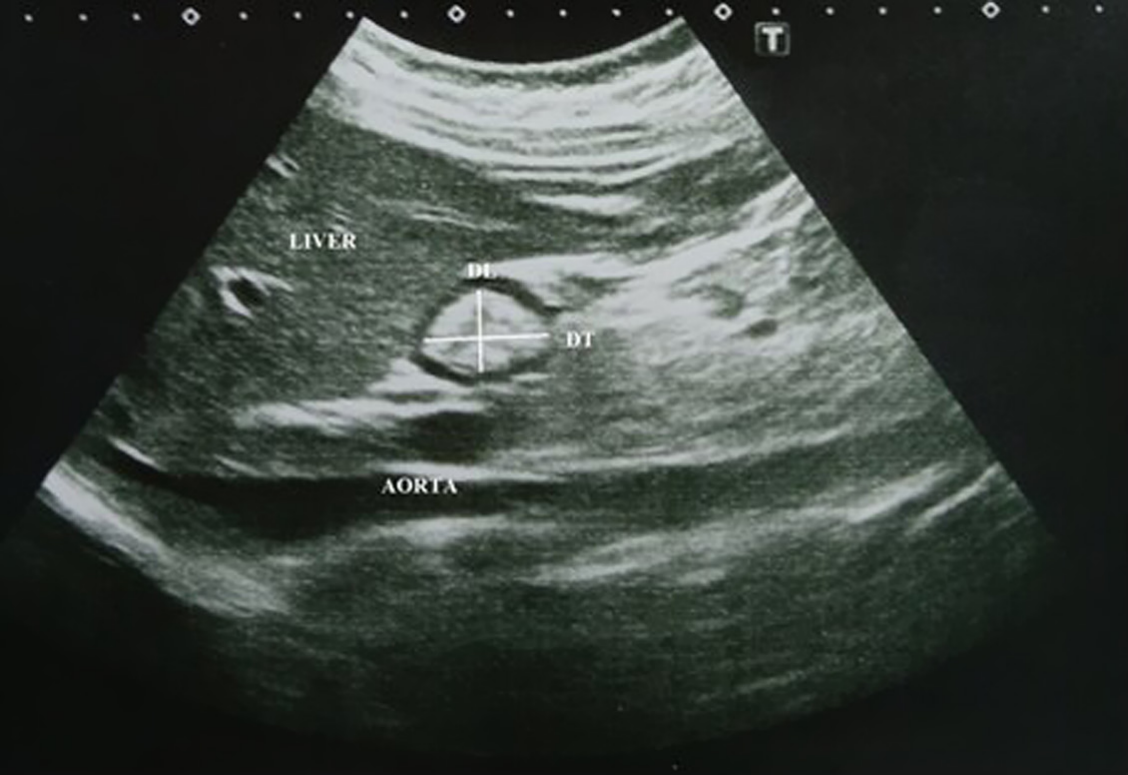
Image of gastric antral cross-sectional area. DL, diameter longitudinal; DT, diameter transversalis.

Cross-sectional area of the stomach antrum was calculated as CSA = (DL × DT × π)/4, where DL is longitudinal diameter and DT is transverse diameter, representing the anteroposterior and craniocaudal sections, respectively.^9^ The gastric antrum was visualized between the left lobe of the liver anteriorly and the pancreas posteriorly at the level of the abdominal aorta or inferior vena cava (Figure 1). The anatomic landmarks mentioned above were used to standardize the scanning protocol.^9,10^

Sonographic measurement of diameters was done by 2 physicians separately for each patient and the images and measurements were checked by a radiologist for every 4 patients. The mean of the 2 measurements for each diameter was used to calculate the CSA. The physicians who performed the US had an experience with 25 cases on sonographic CSA measurement. All sonographic measurements were performed blindly to ingested volume. Cross-sectional area measurements were classified according to image quality as optimal, suboptimal, or impossible. Patients whose measurements were impossible to perform were excluded from the study.

### Statistical Analysis

Categorical data are expressed as percentage and frequency. Continuous parameters were investigated using the Kolmogorov–Smirnov test, histogram, probability plots, skewness, and kurtosis to determine whether they were normally distributed. Descriptive analyses are presented as mean and standard deviation for numeric data with normal distribution and as median and interquartile range for those with non-normal distribution. The relationship between the ingested volume and CSA was examined by Spearman correlation analysis. A multivariate linear regression analysis was done to detect the association between ultrasonographic CSAs and the characteristics of patients including ingested volume, age, height, weight, and BMI. A quantitative model for predicted volume was created by the equation based on the coefficients of significant factors and gastric areas. The capacity of ultrasonographic CSA in predicting the gastric volume at risk for aspiration (volume 0.8 mL kg^−1^) was evaluated using receiver operating characteristic and areas under the curve (AUC). Sensitivity and specificity were determined for cut-off values. A 5% type 1 error level was considered statistically significant for all tests. Statistical analyses were performed using the Statistical Package for the Social Sciences 22.0 statistical package software (IBM Corp.; Armonk, NY, USA).

## Results

A total of 176 patients were investigated in this study. Demographic characteristics and CSA measurements are shown in Table 1. Sonographic images and CSA measurements were of optimal quality at 61% in supine position and 52% in lateral position. Cross-sectional area measurement was impossible to be performed in lateral position in 8 patients (Table 2). 

**Figure 2.**
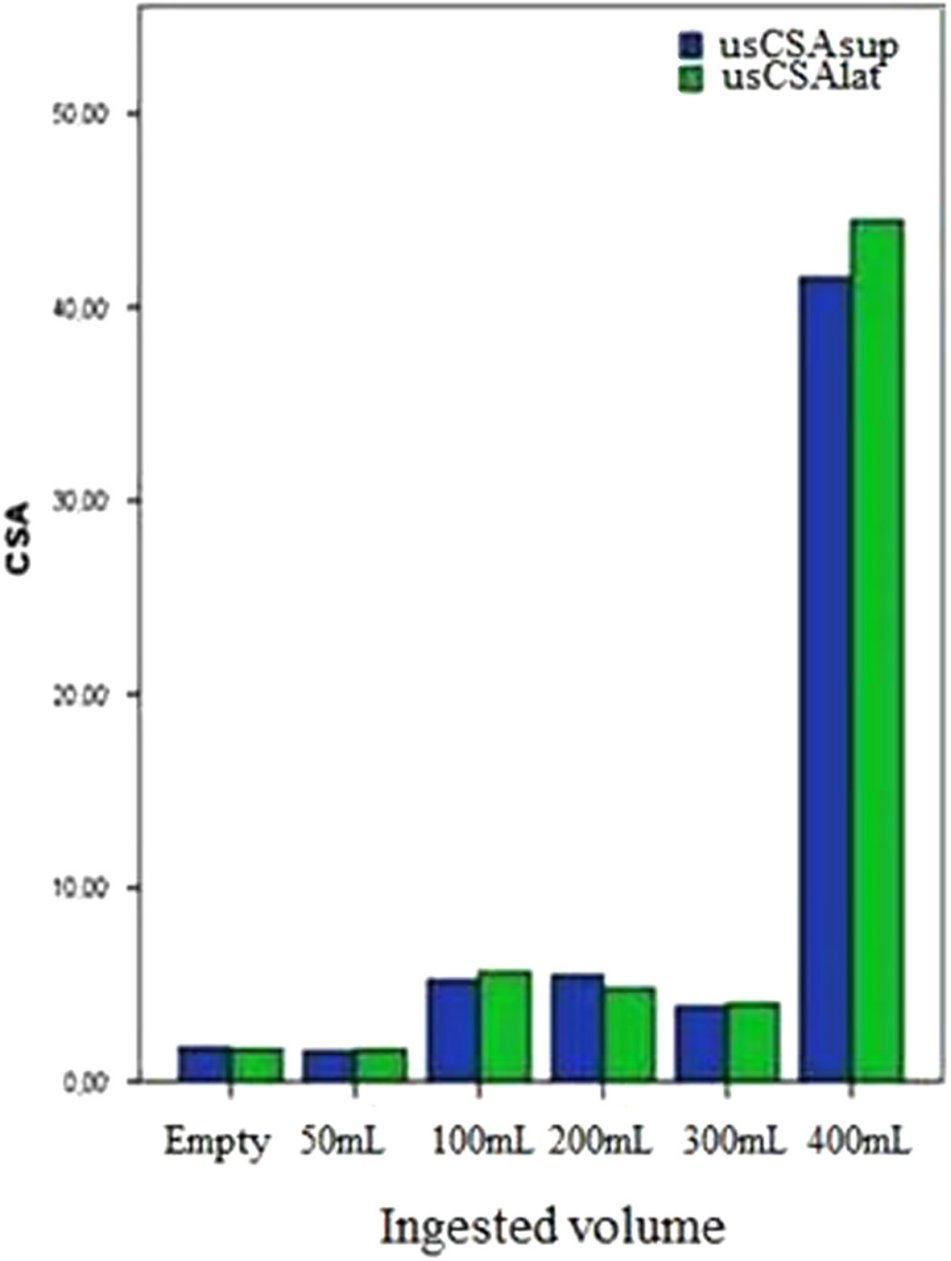
There was a significantly strong linear correlation between CSA and ingested volume up to 200 mL (*r* = 0.781, supine position; *r* = 0.689, lateral position; *P *= .001). However, no consistence could be established between CSA and ingested volume of 300 mL; moreover, disproportionately and sharply high CSA measurements were obtained after ingestion of 400 mL. CSA, cross-sectional area.

The distribution of patients according to ingested volumes and CSA measurements corresponding to ingested volumes are presented in Table 3. There is a significant correlation between ingested volume and gastric antrum CSA up to 200 mL (Figure 2).

Based on the multivariate analysis of significant factors affecting CSA measurement, a quantitative model was determined for predicting gastric volume. Mathematical formula was established using the data of the patients whose sonographic measurement was done at optimal quality. Patients with an ingested volume of 300 mL or 400 mL were excluded. 

As stated in the formula below, the linear regression model showed a significant relationship between predicted volume and BMI in both positions.

Predicted Volume (mL) = 57.3+ 29.4* CSAsup (cm *^2^* ) − 2.6*BMI (kg m **^−^** *^2^* *) (Correlation coefficient=0.787).*

Predicted Volume (mL) = 63.3 + 23.4* CSAlat (cm *^2^* ) − 2.4*BMI (kg m **^−^** *^2^* *) (Correlation coefficient=0.659).*

**Figure 3.**
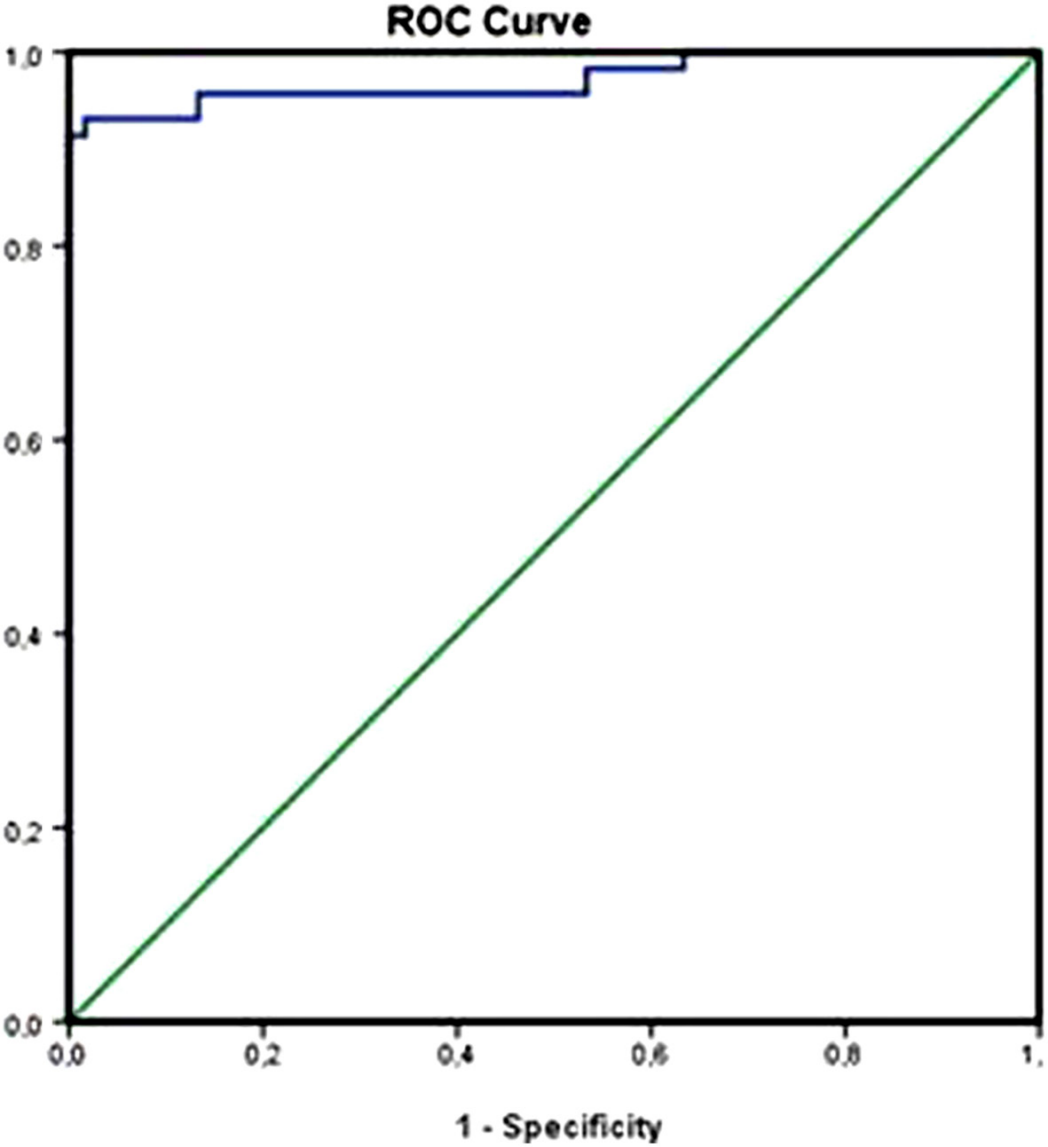
The ultrasonographic CSA cut-off value corre-sponding to a gastric volume above 0.8 mL kg^−g^, which is considered the threshold value for aspiration risk, was 3.08 cm^2^ for supine position. The diagnostic accuracy of these values according to the area under the ROC curves (AUC) were 0.98 (96.2% sensitivity; 100% specificity). CSA, cross-sectional area; ROC, receiver operating characteristic; AUC, area under the curve.

**Figure 4.**
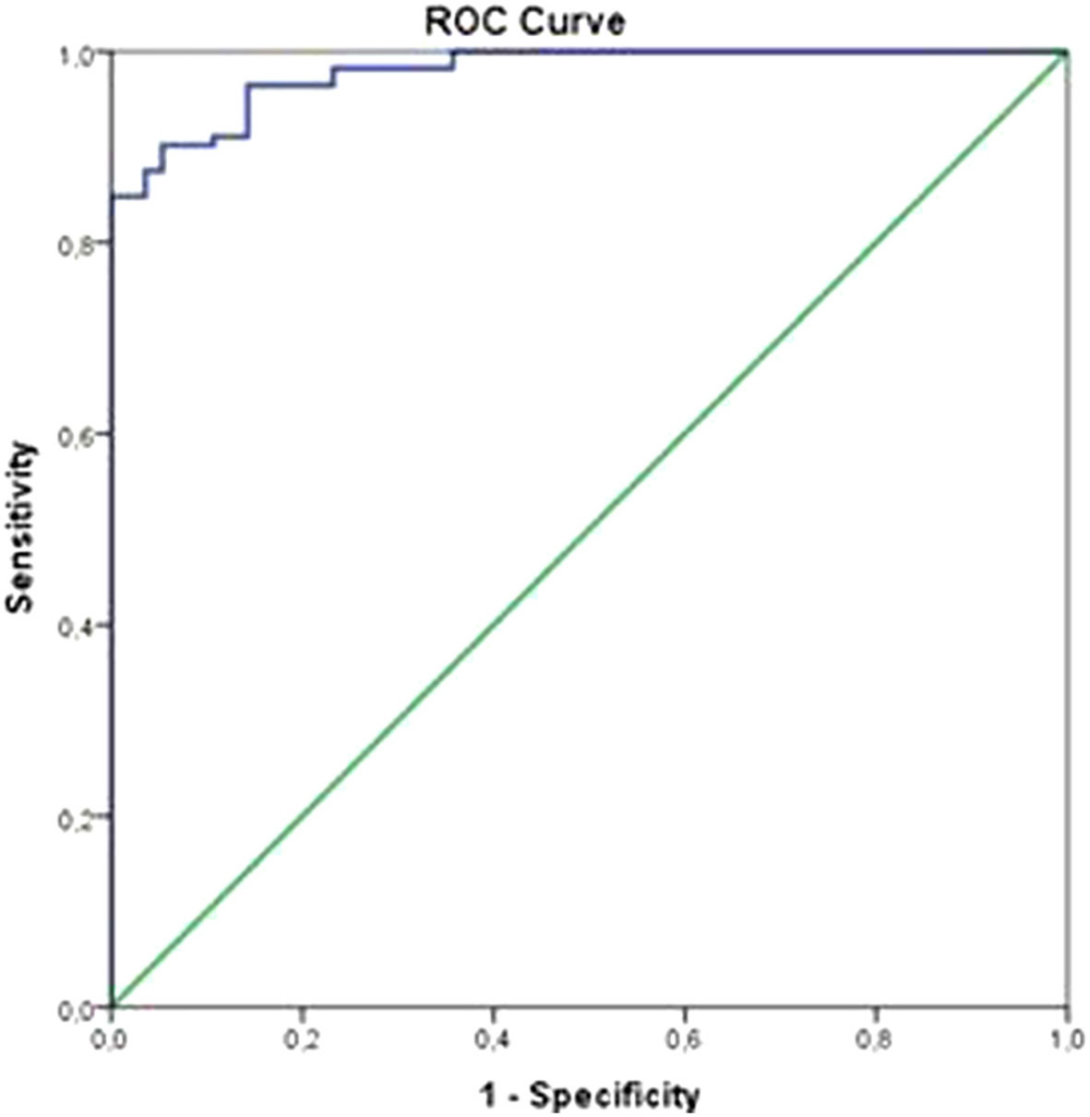
The ultrasonographic CSA cut-off value corre-sponding to a gastric volume above 0.8 mL kg^−g^, which is considered the threshold value for aspiration risk, was 3.09 cm^2^ for lateral position. The diagnostic accuracy of these values according to the area under the ROC curves (AUC) were 0.96 (88.9% sensitivity, 94.9% specificity). CSA, cross-sectional area; ROC, receiver operating characteristic; AUC, area under the curve.

The usCSA values corresponding to risky gastric volume for pulmonary aspiration in either position has been shown in the Figures 3 and 4.

## Discussion

This study investigated the performance of sonographic measurement of antral CSA in predicting gastric volume after ingesting different volumes of a semifluid meal. Thereby, the role of US for diagnosing risky stomach for pulmonary aspiration was also studied.

In this single-center observational study, antral CSA could be viewed in nearly half to two-thirds of patients at optimal conditions according to patient position. This result is in concordance with the data reported by Hamada et al^11^ at 65% in critically ill adults. We could visualize antral gastric CSA in all patients (100%) in supine position, while it was impossible to obtain images in lateral position in 8 patients, corresponding to a 96% yield. 

In early studies, stomach could be identified in up to just 60% of patients by Carp et al^12^ and in 65 to 73% of the time in supine and lateral positions, respectively, by Jacoby et al^13^ at empty stomach. However, current data support our findings with a success rate of 98%-100% in the recent literature.^8,14,15^ Of the 8 patients with impossible measurements, the failure to visualize the gastric antral region was documented at empty state in 4, after an ingested volume of 100 mL in 2, and after an ingested volume of 300 mL in 2. Virtually, the antrum of the stomach is displayed more clearly in lateral position because the gas and stomach contents move towards the antrum under the effect of gravity, making the antrum fuller.^16,17^ We do not know the reason for this unexpected result obtained in the 8 patients, but it may be explained by individual anatomical variations in stomach shape, which should be investigated. 

**Figure 5.**
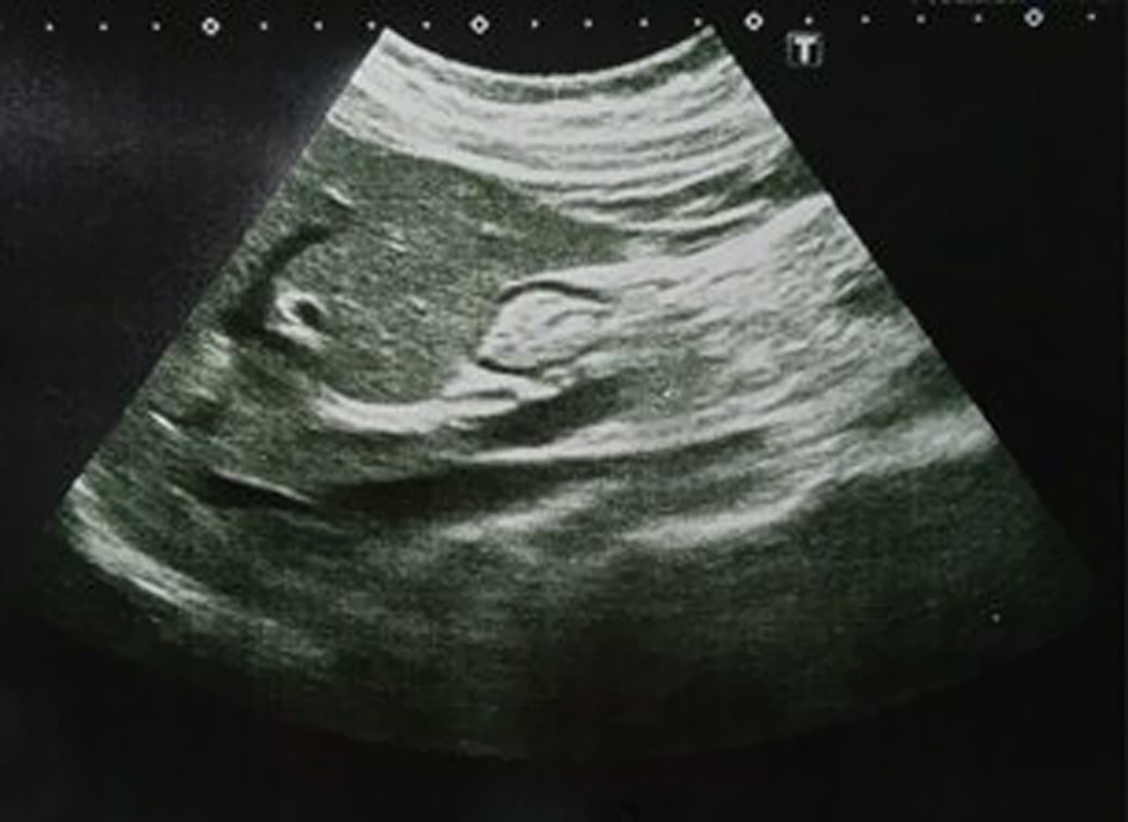
An optimal quality image of antral CSA obtained after ingestion of 100 mL volume in supine position. CSA, cross-sectional area.

**Figure 6.**
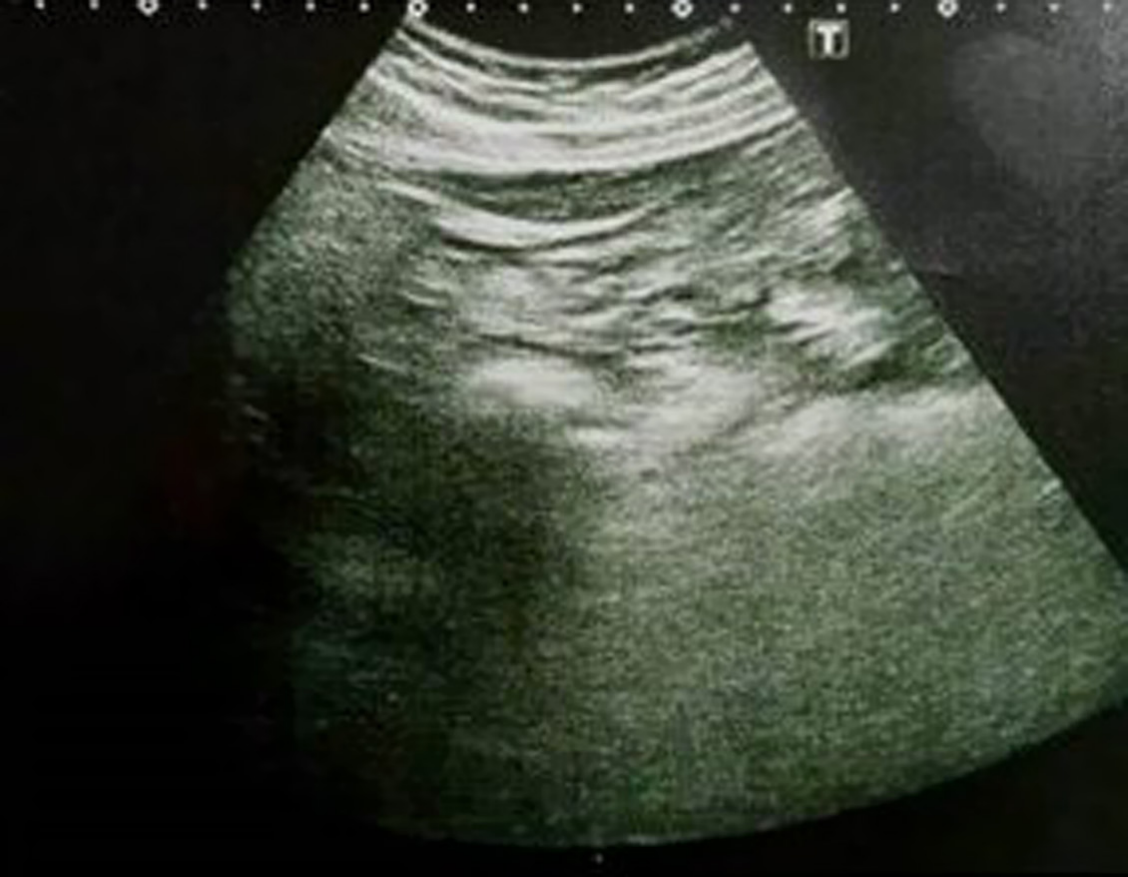
A suboptimal quality image of antral CSA obtained after ingestion of 400 mL volume in supine position. CSA, cross-sectional area.

Measurement of antral CSA using ultrasound has been concluded to be useful for estimating gastric volume in a number of previous studies.^7,18-20^ We detected a linear relationship between CSA and gastric volume up to 200 mL in both positions. However, in contrast to previous reports, the correlation coefficient was stronger in supine position compared to the right lateral position.^8,19,20^ We cannot explain this discrepancy, but it could be due to methodological differences in study design, including patient profile, consistency of ingested volume, and study setting. Increments in ingested volume after the threshold value of 200 mL have been shown to cause an artefactual increase in gastric CSA values, reducing the performance of ultrasound (Figures 5 and 6). The sonographic measurements of all patients with an ingested volume of 400 mL were of suboptimal quality. A similar deviation in linear relation was reported after an ingested volume of 300 mL in Perlas et al.^8^

We report a new mathematical model for each position alternative to the other models reported previously.^7,8,16,20^ The developed predicting model was statistically more accurate in supine position than in lateral position regarding the correlation coefficient (0.79 vs 0.66). The mathematical formula can be applicable to the nonpregnant adult population up to 200 mL gastric volume and 40 kg m^−2^ BMI in supine position. To the best of our knowledge, there are 2 available formulae for predicting gastric volume based on CSA measurement. Of these, the model by Perlas et al^16^ can predict volumes of up to 500 mL, in right lateral position with a correlation coefficient of 0.86, whereas the model by Bouvet et al^7^ is applicable to volumes of up to 250 mL, in semi-sitting position with a correlation coefficient of 0.72.

In the model established by this study, BMI is the only demographic factor with a predictive role on determining gastric volume by CSA measurement. However, the other models were different in terms of the demographic factors affecting the predicted gastric volume. Age was the only demographic factor in the formula of Perlas et al^16^ (Gastric volume = 27.0 + 14.6 * right-lateral CSA − 1.28 * age). The equation proposed by Bouvet et al^7^ included the components of age, height, weight, and physical status [Gastric Volume (mL) = −215 + 57 * log (Antral area (mm^2^)) − 0.78 • age − 0.16 * height (cm) − 0.25 * weight (kg) − 0.80 • American Society of Anesthesiologists Physical Status Classification (1–4) + 16 mL (in case of emergency) + 10 mL (in case of preoperative ingestion of 100 mL antacid prophylaxis)].

Based on the rationale that CSA measurement is correlated with gastric volume, determining a stomach at risk of pulmonary aspiration by measuring gastric CSA has been investigated in different conditions.^7,11^ The minimum gastric fluid volume to induce passive regurgitation of gastric contents and therefore pulmonary aspiration is accepted as 200 mL.^21,22^ However, according to data obtained from animal studies, a gastric volume greater than 0.8 mL kg^−1^ is considered the threshold value for risk of pulmonary aspiration.^3,23^ Bouvet et al^7^ found 3.4 cm^2^ to be a cut-off value for antral CSA at risk of pulmonary aspiration in preoperative setting, with a sensitivity of 91%, a specificity of 71%, and an AUC of 0.90. Another similar study by Hamada et al^11^ reported a cut-off value of 3.6 cm^2^ for risky stomach in critically ill adults with a sensitivity of 76%, a specificity of 78%, and an AUC of 0.80. We found a critical threshold of 3.1 cm^2^ for aspiration risk with a diagnostic accuracy of 96.2% sensitivity, 100% specificity, and 0.98 AUC in supine position. The same value with a close diagnostic performance was also obtained in lateral position.

The main limitation of this study was the research setting and patient profiles. Since the study was confined to the outpatient adult population, the results and the mathematical model cannot be generalized to other settings including critical care, emergency, or perioperative conditions, as well as to patient groups like children, pregnant women, and morbidly obese patients. However, this study was noteworthy for indicating the relationship between ingested semifluid volume and gastric CSA measured by ultrasound. Ingestion of semifluid content, has a similar consistency to gastric juice and a slower gastric emptying compared to the fluid content, which constitutes the strength of the study and its different aspect from most other studies. Also, emptying the stomach completely by endoscopic gastric aspiration before US examination can be considered a methodological advantage of the present study.

## Conclusion

Ultrasonography is a noninvasive and portable tool in predicting gastric volumes up to 200 mL. It can also be used for diagnosing a stomach at risk for pulmonary aspiration before intubation at either elective or urgent conditions. Our findings should be validated with highly qualified studies.

## Figures and Tables

**Table 1.** Demographic Data and CSA Measurements of Study Patients

**Parameter**	**Results**
**Female, N (%)**	93 (52.8)
**Age (years), mean (SD)**	42.7 (15.4)
**Height (cm), mean (SD)**	166 (8.5)
**Weight (kg), mean (SD)**	73.9 (13.4)
**BMI (kg m** **−** **2** **), mean (SD)**	26.9 (5.2)
**CSA sup(cm** **2** **), median (IQR), N(%)**	3.9 (1.9-5.9), 176 (100)
Optimal	3.8 (1.7-5.2), 108 (61.4)
Suboptimal	5.2 (2.6-40.3), 68 (39)
Impossible	Null, 0 (0)
**CSA lat (cm** **2** **), median (IQR), N(%)**	4.2 (1.9-5.8), 176 (100)
Optimal	2.9 (1.7-4.8), 91 (51.7
Suboptimal	5.1 (2.9-40.6), 77 (43.8)
Impossible	Null, 8 (4.5)

CSA, cross-sectional area; sup, supine; lat, lateral; SD, standard deviation; IQR, interquartile range.

**Table 2.** Assessment of Image Quality of CSA Measurements Matched with Ingested Volumes

**Gastric Volume, (mL)**	**CSA Sup Optimal, n (%)**	**CSA Sup Suboptimal, n (%)**	**CSA Lat optimal, n (%)**	**CSA Lat Suboptimal, n (%)**	**CSA Lat Impossible, n (%)**
**Empty **	24 (65)	13 (35)	22 (60)	11 (30)	4 (10)
**50 **	21 (78)	6 (22)	21 (80)	6 (22)	0 (0)
**100 **	22 (85)	4 (15)	18 (69)	6 (23)	2(8)
**200 **	26 (77)	8 (23)	14 (41)	20 (59)	0 (0)
**300 **	15 (58)	11 (42)	16 (62)	8 (31)	2 (8)
**400 **	0 (0)	26 (100)	0 (0)	26 (100)	0 (0)

CSA, cross-sectional area; sup, supine; lat, lateral.

**Table 3.** Cross-Sectional Area Measurements Corresponding to Ingested Volumes

**Ingested Volume**	**CSA sup (cm** **2** **), Median (IQR)**	**CSA lat (cm** **2** **), Median (IQR)**	**N (%)**
**None**	1.8 (1.1-2.4)	1.5 (0.9-2.4)	37 (21.0)
**50 mL**	1.4 (1.2-1.7)	1.7 (1.4-1.9)	27 (15.3)
**100 mL**	5.0 (4.3-5.9)	5.4 (4.3-6.2)	26 (14.8)
**200 mL**	5.3 (4.6-6.7)	4.8 (4.2-5.3)	34 (19.3)
**300 mL**	3.7 (3.2-4.6)	4.1 (2.6-4.8)	26 (14.8)
**400 mL**	41.0 (39.7-43.2)	42.6 (39.4-51.8)	26 (14.8)

CSA, cross-sectional area; sup, supine; lat, lateral; IQR, interquartile range.
